# Periodontal Disease: A Covert Source of Inflammation in Chronic Kidney Disease Patients

**DOI:** 10.1155/2013/515796

**Published:** 2013-06-06

**Authors:** Gener Ismail, Horia Traian Dumitriu, Anca Silvia Dumitriu, Fidan Bahtiar Ismail

**Affiliations:** ^1^Department of Nephrology, Urology, Transplant Immunology, Dermatology and Allergology, “Carol Davila” University of Medicine and Pharmacy, Strada Dionisie Lupu Nr. 37, 020021 Bucharest, Romania; ^2^Department of Periodontology, “Carol Davila” University of Medicine and Pharmacy, Calea Plevnei Nr. 19, Sector 1, 010221 Bucharest, Romania

## Abstract

The prevalence of atherosclerotic complications (myocardial infarction, stroke, and sudden death) is increased in end-stage renal disease (ESRD) patients, especially in haemodialysis patients. Increasing evidence suggests that both in general population and in dialysis patients, systemic inflammation plays a dominant role in the pathogenesis of atherosclerotic complications. In general population, also, evidence shows that moderate to severe periodontitis can contribute to inflammatory burden by increasing serum CRP levels and may increase the prevalence of atherosclerotic events. Moreover, the results of some new interventional studies reveal that effective phase I periodontal therapy may decrease serum CRP levels, the most important acute phase protein, monitored as a systemic marker of inflammation and endothelial dysfunction as well, used as an initial predictor of atherosclerotic events. Considering that moderate to severe periodontal diseases have a higher prevalence in CKD and in dialysis population and that periodontal examination is not part of the standard medical assessment, destructive periodontitis might be an ignored source of systemic inflammation in end-stage renal disease patients and may add to the chronic inflammatory status in CKD.

## 1. Biology and Pathology of Periodontal Diseases

Periodontal diseases represent a group of infectious inflammatory diseases affecting the supporting tissues of the teeth in disease susceptible individuals. The current classification of periodontal diseases, according to the latest one done by AAP consensus meeting in 1999, recognizes plaque-induced gingival disease, early-onset, chronic, and aggressive periodontitis [1]. Plaque-induced gingival diseases (gingivitis) are reversible by treatment as they are limited to the gingiva, different from early-onset, chronic, and aggressive periodontitis which are irreversible forms of periodontal diseases finally causing tooth loss if untreated. Because of great variance in the criteria defining disease, the prevalence of periodontitis is different from one study to another; however, the Third National Health and Nutrition Survey (NHANES III) reported a prevalence of 14% of both moderate and severe periodontitis in the US population older than 20 years of age [2].

The microorganisms that are responsible for initiation and progression of periodontal diseases, along with other bacterial species, colonize the surface of teeth at/or below gingival margin and the epithelial surfaces; in subgingival sites, counts range between 10^8^ in healthy sulci and >10^8^ in deep periodontal pockets [3]. There is a wide variation in the bacterial species profile of subgingival plaque collected from different individuals within a population and from different sites within an individual [4]. In periodontally healthy population, plaque samples consist generally of Gram-positive aerobic species, while in subjects with plaque-induced gingival disease, there is a shift towards Gram-negative species, different *Treponema* species, including the appearance of *Fusobacterium nucleatum*. In plaque samples from chronic periodontitis patients, there is a predominance of Gram-negative species with almost 85% anaerobic or facultative anaerobic species [5].

Among the about 500 different bacterial species that colonize the mouth, only relatively few act as periodontal pathogens; such species are *Actinobacillus actinomycetemcomitans, *serotype a and b,* Bacteroides forsythus *and* Tannerella forsythensis, Campylobacter rectus, Eubacterium nodatum, Fusobacterium nucleatum, Peptostreptococcus micros, Porphyromonas gingivalis, Prevotella intermedia, Prevotella nigrescens, Streptococcus intermedius*, and *Treponema* sp. (*Treponema denticola*) [6].

These organisms reside in biofilms which provide a protective environment against host defence mechanisms as well as locally applied antimicrobials and have specific metabolic properties that are not found in the planktonic state of the bacterial species; consequently, the treatment of periodontal diseases is complex, requiring physical, ecological, and antimicrobial approaches [3].

However, the onset of the disease usually happens after prolonged periods of time from the initial colonization with specific periodontal pathogens, so this seems to be necessary but not sufficient for the progression of periodontal disease [7].

Biofilm formation and persistence on the tooth surface nearby gingival tissues represent a long-lasting challenge to the oral, sulcular, and junctional epithelia. The host epithelial cells are permanently triggered by bacterial enzymes, endo- and exotoxins, metabolic waste products and surface components to secrete proinflammatory cytokines such as TNF*α*, IL-1*β*, IFN-*γ*, and PGE2, and other mediators of inflammation. As inflammation begins, the immune response with both humoral and cell mediated components is either initiated or revived [3]. 

In periodontitis, the inflammatory process extends from the superficial tissues (gingiva) to the deeper connective tissues inducing alveolar bone loss and periodontal ligament destruction as a result of host-derived matrix metalloproteinase synthesis and activation. The ulcerated gingival epithelium migrates apically, lining the periodontal pocket around the affected tooth. The connective tissue adjacent to the periodontal pocket is heavily infiltrated with a dense cellular infiltrate of polymorphonuclear leucocytes, monocytes/macrophages, and B- and T-cell lymphocytes [8]. Waite and Bradley in 1965 [9] estimated that in patients with moderate to severe periodontitis and pocket depths of 6-7 mm and bone loss the surface area of inflammation and infection ranges from 8 to 20 cm^2^, in other words, approximately the size of a palm or larger [10].

Thus, the inflamed and infected periodontal pockets containing highly organized subgingival Gram-negative biofilms can serve as a large reservoir from which the bloodstream is permanently flooded with bacteria, bacterial byproducts such as LPS (lipopolysaccharide), and proinflammatory cytokines that could reach all parts of the body and affect distant sites and organs [11–15].

Additionally, subjects with active periodontal disease or with aggressive forms of disease might have a more responsive immune system than those with inactive disease or chronic forms of disease. Progression of periodontal lesion might expose the host to higher antigenic and inflammatory challenges from active sites where destruction of extracellular matrix is in course, since elevated levels of serum IgG antibody against *Porphyromonas gingivalis* are recorded in subjects with periodontitis and are further increasing with progression of the disease [16].

Subsequently, when assessing the potential role of periodontal disease in systemic inflammation, we might have to take into consideration the severity of disease and to distinguish between progressing forms of the disease and the quiescent ones, not only between periodontally healthy and diseased subjects.

## 2. CKD and Effects on Periodontal Tissues

More and more epidemiologic data support an association between periodontitis and chronic kidney disease (CKD) [17–21]. Recently, Fisher et al. [19] developed multivariable logistic regression models that independently associated periodontitis with CKD and also tested the hypothesis that this might be a bidirectional relationship mediated by diabetes duration and hypertension.

There are well-known effects of CKD and RRT on oral tissues, including xerostomia, delayed tooth eruption, calcification of the pulp chamber, enamel hypoplasia, decreased caries rate, and altered salivary pH values as reported by Davidovich et al. [22] and Proctor et al. [23] in 2005. Also, there are certain effects of CKD and RRT on periodontal tissues. The most common and frequently encountered side-effect is cyclosporine-induced gingival hyperplasia in renal transplantation patients. Increased plaque and calculus levels, gingival inflammation, and probably increased prevalence and severity of periodontal disease in ESRD patients on RRT have also been described. Several studies from Brazil [24], Canada [25], Jordan [26], Israel [22], Spain [27, 28], Taiwan [29], Turkey [30], and the United States [31] have reported increased levels of plaque in HD patients; as a consequence of high levels of plaque, their studies reported increased calculus formation in the same population [22, 24–27, 29, 30] and associated gingival inflammation [22, 24–27, 29–31].

Numerous possible causes have been proposed to explain these findings in CKD patients and in ESRD patients on RRT. Most importantly, we have to consider that these patients are in a state of uraemia which is accompanied by altered immune system because of impaired function of T- and B-lymphocytes as well as monocytes and macrophages [32, 33], resulting in a decreased host response to the subgingival Gram-negative microbial challenge; uraemia might also be accounted for association of increased prevalence and severity of gingival inflammation and periodontitis with increased dialysis vintage [22, 28, 30]. Other studies suggested that CKD patients are less prone to use oral hygiene procedures and to address oral healthcare resources [34–36] because of the intense psychological burden and time-consuming treatment sessions in RRT patients.

Besides uraemia, other contributory factors are the presence of confounding diseases like diabetes mellitus, especially when we take into consideration the high incidence of diabetes in ESRD population and the strong relationship between diabetes mellitus and periodontal disease in general population as reported by Grossi et al. in 1994 [37]; also, secondary hyperparathyroidism in ESRD patients was accounted for alveolar bone loss in renal HD population, but one study on 35 patients failed to find any association between levels of parathyroid hormone and degree of alveolar bone loss or periodontal pocket depth when compared to 35 controls [38].

Additional confounding variables are age, smoking, dialysis vintage, degree of medical management of renal failure complications, access to dental care services, patient recruitment bias, and proper selection of control population [29].

However, there is no data to suggest that the prevalence of moderate and severe periodontal disease in ESRD population is smaller than 14% which is the reported prevalence of the disease in general population [39], but it could be in fact considerably greater.

Moreover, chronic inflammation represents a risk factor for atherosclerotic complications and CKD as well and also plays a role in the pathogenesis of hypertension and diabetes, both being major risk factors for cardiovascular disease and CKD [40]. 

## 3. Systemic Inflammatory Response Induced by Periodontal Diseases and Inferences on CKD

But how might periodontitis influence CKD? Numerous studies have associated periodontitis with increased systemic inflammatory burden, possibly facilitated by acute phase mediators. Analysis of data collected in the NHANES III found a positive correlation between serum C-reactive protein (CRP) levels and the severity of periodontal disease [41]. This finding is supported by later cross-sectional studies [42–45] which also reported the elevation of serum values for CRP and for other mediators of acute phase response such as LDL (low-density lipoprotein) [44, 45], blood glucose [44, 46] and decreased values for HDL (high-density lipoprotein) [43, 44] and peripheral blood neutrophil count [47] and function [48]. 

A study by Rahmati et al. in 2002 [49] on 86 dentate HD patients in USA suggested that periodontitis might be a contributor to systemic inflammation in ESRD patients on HD. Their findings showed that serum levels of IgG against *Porphyromonas gingivalis* and not against other periodontal pathogens were correlated with increased (>10 mg/L) CRP levels which were also associated with lower values of haemoglobin, albumin, iron, total cholesterol, triglycerides, and transferrin saturation. Serum levels of IgG antibody continued to be considerably associated with CRP levels even after adjusting for nonperiodontal causes of increased CRP, haemoglobin, triglycerides, and transferrin saturation values.

A study from Spain by Castillo et al. in 2007 [28] found that HD patients had a greater number of periodontal pathogens than the controls.

Chen et al. in 2006 [29], in a study of a large sample of ESRD population on HD, found that the prevalence and the severity of periodontal disease were greater in general population, this data being supported by two other studies from the USA [31, 50]. Another important finding was that severity of periodontitis independently correlated with age, smoking status, diabetes, low albumin levels, and dialysis vintage.

In a retrospective study of mortality among haemodialysis patients, periodontitis increased the risk of cardiovascular events related death by five times, after adjusting for other important associates of CKD [51].

So, the hypothesis that periodontal disease might be a contributing factor to CKD through the systemic inflammatory response which is accompanying it is biologically plausible.

Remarkably, several studies (Table 1) report that effective initial periodontal therapy, consisting only in local scaling and root planning and microbial biofilm control, might reduce systemic markers of inflammation, such as haptoglobin, in periodontal patients, whereas addition of NSAIDs, like flurbiprofen, to initial periodontal therapy was shown to reduce both haptoglobin and CRP levels [52]. Studies of D'Aiuto and coworkers reported a decrease of both CRP and IL-6 and of LDL cholesterol at 6 months after initial periodontal therapy, results which remained significant after adjusting for age, smoking status, body mass index, and gender [53–55]. Tonetti et al. in 2007 [56], in a study on 61 patients diagnosed with severe generalized periodontitis have reported that intensive periodontal therapy, consisting in supra- and subgingival scaling and root planning, placement of microspheres of minocycline, and extraction of hopeless teeth, resulted in a transient, acute short-term inflammatory response and altered endothelial function, but at 6 months, the study group showed better endothelial function than the control group.

The most recent study of Wehmeyer and coworkers, in 2013 [57], conducted an investigation on 53 ESRD patients (26 in the treatment group and 27 in the control group) with associated moderate and severe chronic periodontitis; intensive periodontal therapy was delivered and the following parameters were measured: clinical periodontal indices, serum albumin, and IL-6; at 3-month postintervention, there was a statistically significant improvement of the periodontal parameters for the treatment group, but at 6 months, the difference was no longer present; however, serum albumin and IL-6 showed no significant difference between groups, at any time, during the follow-up period.

In conclusion, periodontitis might increase serum CRP levels and other serum inflammatory markers, but efficient periodontal therapy could decrease CRP values.

All the discussed facts are of importance for both ESRD patients on RRT (haemodialysis patients or peritoneal dialysis (PD) patients) and nondialyzed CKD patients, considering that 30%–50% of these/subjects have evidence of active systemic inflammatory response with elevated levels of serum CRP and other proinflammatory cytokines (IL-1, IL-6, and TNF-*α*) [58–61].

A chronic, low-grade inflammatory status is a usual phenomenon also found in patients with early stages (2 to 4) of CKD [62–65]. Although there are multiple causes for elevated CRP levels in ESRD population, many patients experience high serum CRP values without any visible signs of infection and/or inflammation. The causes of inflammation are not entirely understood, but lately, it has become evident that inflammation is one of the strongest predictors of poor clinical outcome in these patients [66].

So, in light of all these data, we can hypothesize that periodontitis, in its moderate and severe forms, might represent one of the sources of systemic inflammation, one of which is possible to manage through efficient initial periodontal therapy.

## 4. Conclusions

Periodontal disease in its moderate to severe forms is prevalent in general population, and it might be more prevalent in predialysis CKD patients and in ESRD patients on RRT. 

Furthermore, periodontitis has been correlated with elevated levels of systemic inflammatory markers. 

A limited number of studies conducted both in general population and in CKD patients reported that efficient initial periodontal therapy might lower serum levels of some pro-inflammatory biomarkers. 

However, more interventional studies on larger groups are necessary to evaluate whether treatment of periodontal disease will result in decreased all-cause and cardiovascular mortality within these patients and whether by reducing systemic inflammatory burden the progression of the renal disease will also be slowed down.

Taking into consideration the following facts that all ESRD patients on RRT are potential candidates for kidney transplant, the possibility in predialysis CKD patients to slow the progression of the renal disease by reducing systemic inflammatory burden, and the potential contribution of periodontal disease to the inflammatory status in CKD patients, it seems important to evaluate and preserve periodontal tissue health of this population.

## Figures and Tables

**Table 1:** Interventional studies (impact of periodontal therapy on systemic inflammatory biomarkers).

Study	No. of patients	Type of study	Serum inflammatory biomarkers	Results
Ebersole et al., 1997 [52]	34 pts/AP, 35 pts/ctrl	Prospective/2 yrs interventional, SRP at every 6 mos + Fb (5,15 or 50 mg/b.i.d.)	CRP, Hp	↓ CRP, Hp at 2 yrs
D'Aiuto et al., 2004 [53]	94 pts/SGP	Prospective/6 mos interventional, SPT (SRP)	CRP, IL-6	↓ CRP, IL-6 at 6 mos
D'Aiuto et al., 2005 [54]	65 pts/SGP	Prospective/2 mos interventional, IPT (SRP + Local Ab)	CRP, IL-6, LDL chol total chol	↓ CRP, IL-6, ↓ LDL chol ↓ total chol at 2 mos
D'Aiuto et al., 2006 [55]	40 pts/SCGP	Prospective/6 mos interventional randomized SPT/IPT	CRP, IL-6, total chol	↓ CRP, IL-6 ↓ total chol at 2 and 6 mos in both groups but more important in the IPT group
Tonetti et al., 2007 [56]	61 pts/SGP	RCT/Prospective/6 mos interventional IPT	CRP, IL-6 sE-selectin	transient, acute, short-term ↑ CRP, IL-6 ↓ CRP at 6 mos ↓ sE-selectin
	59 pts/ctrl	SRP		

AP: adult periodontitis; SGP: severe generalized periodontitis; SCGP: severe chronic generalized periodontitis; SRP: scaling and root planning; SPT: standard periodontal therapy; IPT: intensive periodontal therapy; Fb: flurbiprofen; Ab: antibiotic; CRP: C-reactive protein; IL-6: interleukin-6; LDL chol: LDL cholesterol; Hp: haptoglobin; sE-selectin: soluble E selectin; yrs: years; and mos: months.
